# Assessment of the national and subnational completeness of death registration in Nepal

**DOI:** 10.1186/s12889-022-12767-z

**Published:** 2022-03-04

**Authors:** Surender Prasad Pandey, Tim Adair

**Affiliations:** 1 Melbourne School of Population and Global Health, University of Melbourne, Melbourne, Carlton, VIC 3053 Australia; 2 Ministry of Federal Affairs and General Administration, Kathmandu, Nepal

**Keywords:** Nepal, Civil Registration, Vital statistics, Death registration, Online registration, Completeness of registration, Mortality, Subnational

## Abstract

**Background:**

Reliable and timely mortality data from a civil registration and vital statistics (CRVS) system are of crucial importance for generating evidence for policy and monitoring the progress towards national and global development goals. In Nepal, however, the death registration system is not used to produce mortality statistics, because it does not providing data on age at death and only reporting deaths by year of registration. This study assesses the completeness of death registration in Nepal – both the existing offline system and the newer online system – as well as the completeness of death reporting from a CRVS Survey, and assesses differences by year, sex, ecological belt, and province.

**Methods:**

The empirical completeness method is used to estimate completeness at all ages from the offline (paper-based) registration system (2013-17), the online registration system (2017-19) and the CRVS Survey (2014-15).

**Results:**

Completeness of the offline death registration system was 69% in 2017, not increasing since 2013 and being higher for males (73%) than females (65%). Completeness of online registration was only 32% in 2019, but almost double the 2017 figure. Completeness of death reporting in the CRVS Survey was 75% in 2015. The largest subnational differentials in completeness exist for the offline registration system, ranging from 90% in Gandaki to just 39% in Karnali.

**Conclusions:**

Improvement in the utility of the Nepalese death registration system for mortality statistics is dependent on continued roll-out of the online death registration system (which reports age at death and deaths by year of occurrence) throughout the country, focusing on areas with low registration, building a strong coordination mechanism among CRVS stakeholders and implementing public awareness programs about death registration.

**Supplementary Information:**

The online version contains supplementary material available at 10.1186/s12889-022-12767-z.

## Background

Routine, timely and accurate mortality data should be a primary source of evidence on population health for policymakers. However, globally around two-fifths of deaths remain unregistered, meaning that health policy is dependent on suboptimal evidence due to incomplete and poor-quality mortality data [1]. Due to this health information gap and persistent policy dependence on unrealistic information, desired progress to improve public health is challenged [2]. Like many other low- and middle-income countries, existing routine mortality data sources in Nepal suffer from the issues of completeness and quality, and the availability of quality mortality data is ranked as being suboptimal by different data assessment studies [3, 4]. Estimates of mortality for Nepal made by Central Bureau of Statistics (CBS), Global Burden of Disease (GBD) Study and United Nations World Population Prospects (UN WPP) are largely reliant on early age mortality data from the Demographic Health Survey (DHS), Population Census and Multiple Indicator Cluster Survey (MICS) and is heavily reliant on modelling based on other countries and socio-economic covariates, with the no local data from routine sources used [5–7].

A complete and well-functioning civil registration and vital statistics (CRVS) system is the best option for supplying continuous and uniform mortality data to provide evidence of progress towards local, national, and international health goals [8]. Due to this inherent utility of the CRVS system as a source of mortality, CRVS system strengthening has been a global and national priority in recent years, as demonstrated by various national, bilateral, multilateral and philanthropic initiatives [2, 9]. As a member state of the UN, Nepal is committed to adopting UN recommendations and regional action frameworks for strengthening national the CRVS system as the source of vital statistics and its utilization for effective and regular monitoring of the progress towards development goals. The Department of National ID and Civil Registration (DONIDCR) is the sole national CRVS entity, which manages both online and offline civil registrations, compiling reports from Local Registrar’s Offices, and publishing and disseminating annual civil registration reports. At the subnational level, municipal wards are the major implementing agencies in the form of Local Registrar’s Offices. At the local level, ward level officials act as local registrars under dedicated authority from the national Civil Registrar and send periodic progress reports to the DONIDCR, which form the basis of annual civil registration reports compiled and published by DONIDCR. The major CRVS stakeholders are the Ministry of Health and Population, Central Bureau of Statistics, Ministry of Home Affairs (DONIDCR), UNICEF and the World Health Organization (WHO).

However, despite the CRVS system being implemented officially in Nepal since 1976, its major focus has been for developing legislative or administrative components, with inadequate emphasis on vital statistics [10, 11]. CRVS stakeholder coordination is limited to the legislative component of the system and with no focus on vital statistics [10]. As a result, the Nepal CRVS system is poorly functioning and incomplete and has primarily been used for the purposes of registration of deaths rather than for mortality statistics; no adequate reliable and timely mortality statistics have been compiled using this source [10]. Most deaths are reported using a traditional paper-based system by the Local Registrar’s Offices that only provides aggregate data without age at death. The CRVS system also does not collect reliable cause of death data; the death notification form has a section to report the cause of death but this is not based on the International Form of Medical Certificate of Cause of Death and more than 75% of deaths are reported simply as a ‘natural death’. There is hence a major need in Nepal to make greater use of its routine CRVS system to provide timely mortality estimates at the national and subnational (local) levels.

A recent encouraging development has been the introduction of an online component of the CRVS system, which regularly reports deaths at the individual level with data on age at death. The establishment of the online registration system by DONIDCR at local levels is gradually being extended and reached approximately 40% population coverage by 2020 [12]. Importantly, incomplete death registration data that are adjusted based on their estimated completeness can be used to estimate key mortality indicators, as they are by the GBD and UN WPP [6, 13]. The only other source of routine mortality data is the Health Management Information System (HMIS), which only reports data on 10,726 deaths that occur in hospitals, or 6% of estimated annual deaths [14].

Other mortality data sources, including census and surveys, do not provide routine and disaggregated mortality statistics [8]. Data on mortality at all ages have been collected in ad-hoc surveys such as the CRVS Survey of 2015 or the Post-Earthquake Survey conducted after the 2015 earthquake [15]. However, their irregular or ad hoc nature limit their utility [5]. The DHS, conducted most recently in 2016, estimated adult mortality using the sibling survival method, which is subject to recall and selection bias, although methodological developments have resulted in improved estimates from this source [16–18]. The Population Census of 2011 collected information on household deaths in the previous 12 months, however such estimates are also affected by incompleteness and this source is only conducted every 10 years. Although there have been several surveys that have collected data to enable estimation of child mortality, the limitations of traditional surveys and censuses include challenges to obtain disaggregated small area estimates, lack of cause of death information and an inability to estimate mortality at adult and older ages [5]. In addition to these issues, censuses and surveys are relatively expensive to conduct compared with a CRVS system.

In this context, death registration data from the CRVS system is the most feasible data source to generate reliable and routine mortality estimates in Nepal across all ages at both the national and subnational levels. Subnational estimates are particularly important in Nepal given the considerable socio-economic diversity of its population [19, 20]. Before the CRVS system is used for these purposes, there is a need to investigate its completeness and quality, which will also provide evidence for interventions to improve the system and also to adjust data to produce reliable mortality statistics. The first objective of this study is to assess the completeness (i.e. the percentage of actual deaths that are registered) of the death registration system (both paper and online) by sex, year and province and ecological belt. For comparison, especially at the subnational level, the study also assesses the completeness and quality of mortality reporting in the CRVS Survey.

## Methods

### Mortality data sources

A summary of mortality data sources in Nepal is shown in Additional File 2: Table A1.

### Civil Registration and Vital Statistics (CRVS) system

The CRVS system of Nepal was officially introduced in the 1970s after promulgation of the ‘Birth, Death and Other Personal Event Registration Act 1976’ and ‘Birth, Death and Other Personal Event Registration Regulations 1977’. The Constitution of Nepal 2015 retained civil registration as the common responsibility of the three level of governments in Nepal: The Federation, Provincial and Local Governments. The DONIDCR, under the federal Ministry of Home Affairs, bears the overall management of the national CRVS system and the local ward offices are the major implementing agencies. The Director General of the DONIDCR acts as the Civil Registrar of Nepal. Local registrars are deployed at the local (ward) levels and have a crucial responsibility to register events, keep records and report personal events to the DONIDCR. The CRVS system at newly formed provincial levels is still not clearly defined and implemented but is maintained under the Social Development Ministry. There are two registration systems: the existing offline (paper based) system that has been used for decades and the online Management Information System (MIS), which was introduced in 2015.

Deaths occurring in both communities and facilities are notified by the next to kin of the deceased to the Local Registrar’s Office and have been registered by the local registrar either using the local register (i.e. offline system) or using the online system (where available). The Local Registrar’s Offices keep the records (including notification forms) and send monthly and annual reports of aggregated deaths to the DONIDCR (offline registration) or continuously update the online MIS system. The offline registration system lacks variables important for generating vital statistics, including date and place of occurrence and age of deceased. Although age at death is collected in the death notification forms, it is not compiled in a database in the offline system because local registration offices only report aggregate number of registered deaths by sex, and so only total death numbers are available at the national, provincial and ecological belt levels. The online registration system however includes key information about deaths, including date of death, place of occurrence, and date of registration, but far fewer deaths are presently being registered by the online system, especially in rural and remote areas [20]. For instance, in 2017, only 23,412 deaths were registered in online system, which were only 20% of the number of deaths (114,436) registered offline in the same year [21, 22]. Some deaths may appear in both offline and online systems due to reporting errors; for example, some deaths which have already been registered in the offline system may be re-entered in the online system at the time of issuance of a duplicate copy of the death certificate.

### CRVS Survey

A nationally representative CRVS Survey was conducted by CBS on behalf of the Department of Civil Registration (DOCR) in 2015/16 to collect the baseline data on the overall status of civil registration in the country. The survey collected reported household deaths over three years (with the other five events, including birth, marriage, divorce, migration, and adoption), as well as the registration status and time of registration after the occurrence of these deaths, among other information on registration access [15]. In this survey, a total of 80,000 households were interviewed from 1,600 enumerations area, and 4,532 deaths were collected from the sampled households, implying an estimated national total of 306,073 deaths during the reference period. Out of those deaths, 73,704 (24.1%) were reported to have occurred in 2013/14, 92,974 (30.4%) in 2014/15 and 139,395 (45.5%) in 2015/16 [23]. A plausible reason for the higher percentage of reported deaths in the more recent years of reference period is due to a greater likelihood of omission of deaths by respondents in earlier years. The CRVS Survey reports that about three-quarters of the deaths reported were stated by the respondent as having been registered. However, we do not analyse subnational differences of self-reported registration status in this study. This is because self-reported completeness is not directly comparable to the other estimates of completeness in this study that are estimated based on data produced in the offline or online registration system or from reports of actual deaths in the CRVS survey. Self-reported completeness relies on accurate reporting of death registration by respondents, but it may be biased if the respondents over-report registration because they are concerned about receiving a penalty if they state that a death has not been registered. Self-reported completeness may also be biased because it is only reported for those deaths identified in the CRVS survey (i.e. not all deaths), which is particularly an issue for earlier years where the completeness of CRVS Survey death reporting was lower. The self-reported registration data will be analysed in further detail in a future study because they provide valuable data on socio-economic differentials in registration and reasons for registration and non-registration. Further details about the CRVS Survey sample design are provided in Additional File 1.

The CRVS Survey data were published according to the Nepalese calendar year, which starts in mid-April. Hence, to make data comparative we converted Nepali calendar data into the Gregorian calendar year are referred to as 2014/15, 2015/16 etc. (see Additional File 1).

We received written approval from DONIDCR and CBS to use the data produced by both agencies for this study.

### Assessment of completeness

We calculated the completeness of each mortality data source at all ages (that is, the percentage of actual deaths that the source registers or reports) using the empirical completeness method [24]. The empirical completeness method was developed from approximately 2,500 country-years of data from over 100 countries and estimates completeness by sex based on the key drivers of the crude death rate – an estimate of the true under-five mortality rate (*5q0*; number of deaths under the age of five years per 1,000 live births) and the percentage of the population aged 65 years and above – as well as the registered crude death rate (the number of registered or reported deaths per 1,000 population). This is relatively straightforward to implement and relies on data that are commonly available at the national and subnational level. The method overcomes the limitations of existing direct (capture-recapture) and indirect (death distribution methods) completeness methods which lack accuracy and timeliness, especially at the subnational level, are complex to apply and, in the case of indirect methods, require data on age at death [24].

One limitation of the empirical completeness method is that it is not designed to accurately measure completeness where there has been a mortality shock, such as a natural disaster like an earthquake (which is relevant for Nepal – see below) or where HIV deaths and adult mortality is unexpectedly high for given *5q0* levels (which is used in the method to represent overall mortality). The empirical completeness method has been previously used to estimate completeness for 2,844 counties in China [25]. We used Model 2 of the method, because we could not use Model 1 which relies on completeness of under-five death reporting, which cannot be calculated for the offline registration data because it does not have age at death data. Also, it is not possible for the empirical completeness method to produce completeness estimates of above 100%, which can occur if there are high levels of late registrations, because it is based on a logit model, however completeness is not close to 100% in any subnational area of Nepal. Completeness was estimated nationally, for each province and each ecological belt. Ecological belts and provinces were chosen to assess the quality and completeness of mortality data at subnational levels and ensure the sensible representation of geographical and political areas of Nepal. All these subnational areas have extensive socio-economic and ecological diversity, which significantly impact the mortality levels, patterns, and completeness. The under-five mortality rate was estimated from the United Nations Inter- Agency Group for Child Mortality Estimation (IGME) [26] data and subnational estimates from the census and DHS [27]. To assess sensitivity of the provincial completeness estimates to the under-five mortality rate used, we also estimated completeness using the IGME estimate of the under-five mortality rate for each province for both sexes.[26] Three separate population estimates (census, UN WPP and GBD) [7, 28, 29] were used as input data at the national level to assess the sensitivity of completeness estimates to the source of population data, while subnational population was projected based on CBS estimates (see Additional File 1). We also calculated completeness of offline registration compared to the estimated deaths of the GBD Study 2019 and UN WPP 2019.[6, 13] Further detail of their methods is shown in Additional File 1.

In April 2015 a major earthquake struck Nepal, killing an estimated 9,000 people, predominantly in Bagmati province and the Mountain and Hill ecological belts. Given that the empirical completeness method cannot be used in settings where there has been such a mortality shock, we did not attempt to calculate completeness in these provinces for the CRVS Survey or for 2015 for the offline registration data. For the offline registration data in 2016, estimates for Bagmati and Hill were based on the figures for Nepali year 2073 (April 2016-April 2017), while estimates were not made at all in Mountain region because of an unusually high number of deaths likely due to late registrations from the previous year when the CRVS system was affected adversely by the earthquake.

## Results

### Offline registration

**Fig. 1:**
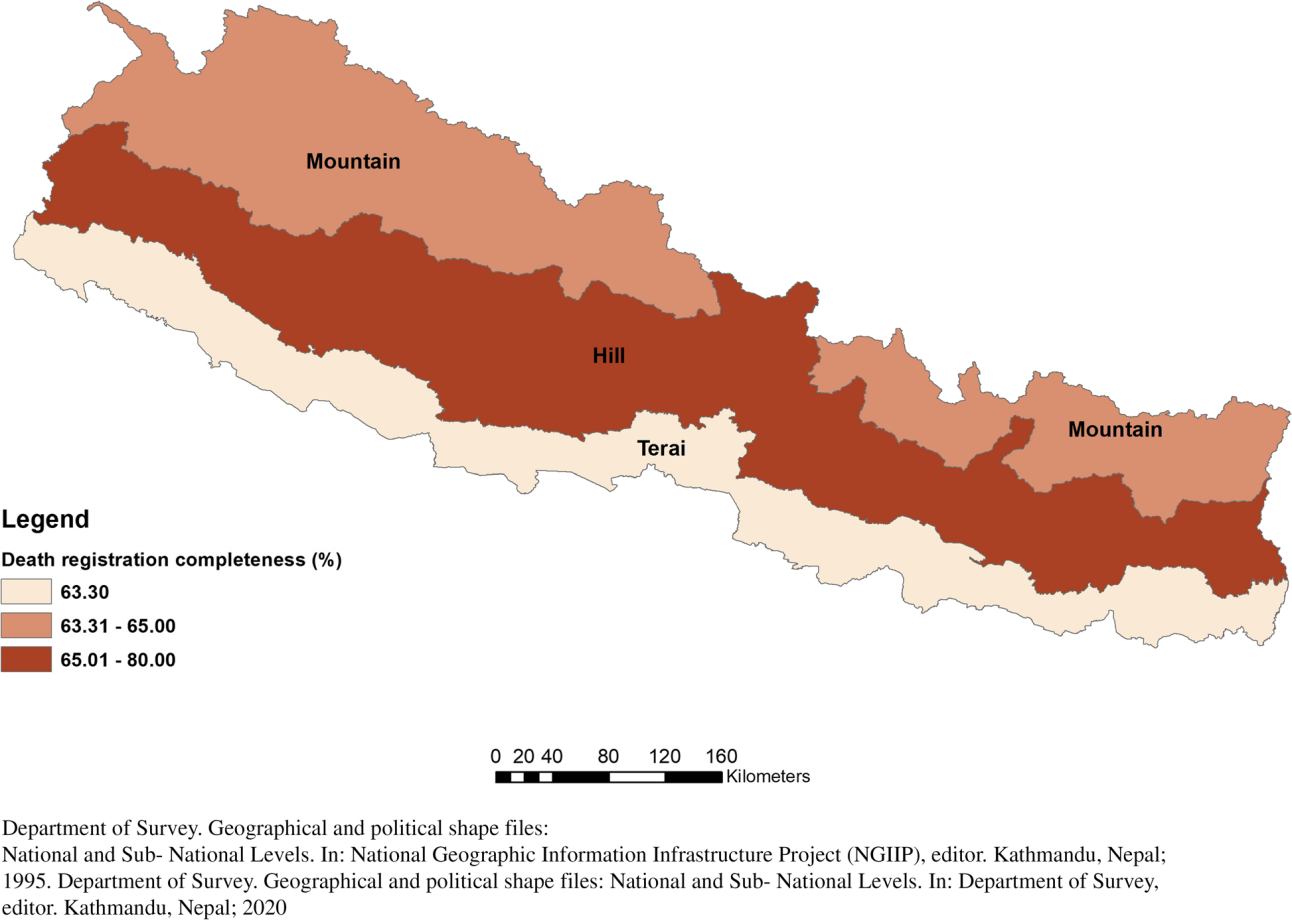
Death registration completeness (offline) by ecological belts, 2017

**Fig. 2:**
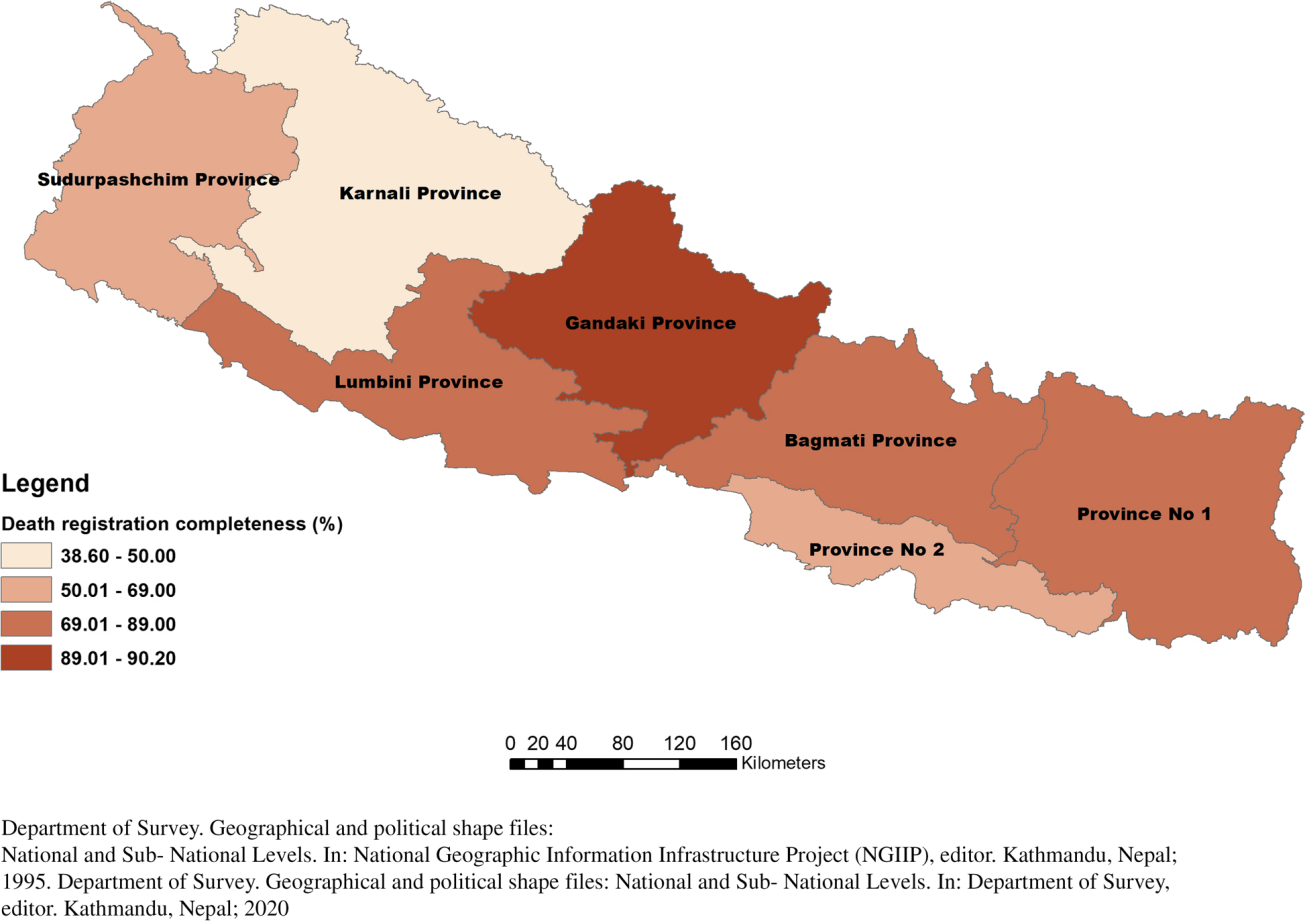
Death registration completeness (offline) by provinces, 2017

Application of the empirical completeness method estimates that the offline death registration system was 69% complete in 2017 (Table 1). There was no trend of increasing completeness of offline death registration in Nepal from 2013 to 17, with estimates ranging from 71% to 2013 to 62% in 2015. Death registration completeness for males was consistently higher than for females (2017: 73% males, 65% females). Among the ecological belts, Hill has the highest offline registration completeness, reaching 78% for both sexes in 2017, followed by Mountain (65%) and Terai (63%) (Figs. 1 and 2). By province, the highest completeness of offline registration in 2017 was found in Gandaki (90%), followed by Lumbini (78%), Bagmati (77%), and Province 1 (75%), with the lowest completeness in Karnali (39%) and Province 2 (54%), which were each below 50% for two years in the period. The provinces, overall, each had consistent levels of completeness of registration over the period, aside from lower completeness in Province 2 in 2015 and 2016 and in Province 1 and Karnali in 2017. There was a particularly large difference in completeness in Sudurpashchim between males (77%) and females (46%). Spatially, there is no clear pattern to offline death registration levels, as shown in Figs. 1 and 2. Use of the IGME provincial under-five mortality rates results in exactly the same ranking of completeness by province, with the difference being less than 1% point in three provinces and reaching 7.6% points in Sudurpashchim (Additional File 2: Table A3). Overall, completeness estimated using UN WPP and GBD population estimates resulted in slightly higher completeness according to the UN WPP population and slightly lower estimates using the GBD population, but the differences were generally within only 2% points.

**Table 1:** Summary of All-Age Completeness (%), Offline Registered Deaths (2013-2017)

	Offline registration																		
	**2013**				**2014**				**2015**				**2016**^a^				**2017**		
**Area**	Both	Male	Female	Both		Male	Female	Both		Male	Female	Both		Male	Female	Both		Male	Female
**Nepal (Census Projected Population)**	71.1	76.0	64.2	70.7		73.7	66.7	62.0		66.6	57.5	67.9		73.6	61.0	69.4		73.0	65.4
**Nepal (UN WPP population)**	72.6	78.6	64.5	72.8		77.7	67.1	64.7		71.7	58.0	70.7		78.8	61.5	72.1		78.2	65.6
**Nepal (GBD Population)**	69.9	74.6	63.8	69.4		72.4	66.1	60.8		65.3	56.9	66.4		72.3	59.9	67.7		71.5	63.7
***Ecological Belts***																			
**Mountain**	54.5	62.5	45.2	61.8		69.4	51.6	-		-	-	-		-	-	64.9		72.9	53.9
**Hill**	76.3	80.3	70.6	77.3		79.4	74.2	-		-	-	76.6		81.9	69.0	78.1		81.0	74.0
**Terai**	70.6	75.3	64.1	67.6		70.5	64.4	55.6		59.7	52.4	62.0		66.3	58.0	63.3		66.0	61.1
***Provinces***																			
**Province 1**	83.9	86.1	80.1	84.4		85.9	81.8	83.4		85.3	80.6	82.7		85.6	78.4	74.6		77.0	72.7
**Province 2**	59.7	64.0	55.7	52.8		55.4	51.7	28.4		29.4	30.8	40.2		41.6	42.1	54.1		55.9	54.1
**Bagmati**	73.9	76.3	70.3	72.3		73.2	70.8	-		-	-	78.4		80.2	75.6	77.1		78.3	75.2
**Gandaki**	83.5	87.2	77.9	88.8		90.6	85.3	85.1		88.2	80.3	87.0		90.2	81.6	90.2		91.6	87.5
**Lumbini**	80.0	83.2	74.7	79.8		80.4	78.6	77.3		81.2	71.9	75.5		80.6	68.6	77.7		80.1	74.6
**Karnali**	45.5	51.7	41.0	55.1		60.2	50.9	50.0		56.3	45.1	50.3		59.9	41.3	38.6		42.9	37.3
**Sudurpashchim**	68.9	81.5	48.9	61.6		73.8	46.4	52.7		67.0	37.1	67.5		83.1	41.0	63.8		76.8	45.8

^a^ Data for Hill and Bagmati are for year 2073 (April 2016-April 2017)

A comparison of the completeness estimates for Nepal for 2017 with annual deaths estimated by UN WPP 2019 and the GBD Study 2019 are presented in Table 2 [6, 13]. Comparatively the completeness of offline registered deaths in 2017 (69.4%) is higher than completeness calculated according to either UN WPP (63.8%) or GBD (60.7%) estimated deaths. Male completeness according to the UN WPP was 20% points higher than female completeness, with the latter 10% points lower than estimated by the empirical completeness method. However, according to GBD estimated deaths the difference between male and female completeness is similar to the empirical completeness method.

**Table 2:** UN and GBD estimated death completeness (%), Nepal, 2017

Method	Both sexes	Male	Female
Empirical completeness method	69.4	73.0	65.4
UN WPP estimated deaths	63.8	73.6	53.4
GBD estimated deaths	60.7	65.6	54.6

### Online death registration completeness

Table 3 demonstrates that online death registration completeness has grown from 18.6% to 2017 to 25.7% in 2018 and 31.9% in 2019. Completeness was very similar for males and females. Again, completeness when using the GBD and UN WPP estimated population was very similar to when using the Census based projected population. At the subnational level, each ecological belt had much higher completeness in 2019 than the previous two years, with each being above 30% in 2019. Completeness in Mountain increased sharply, rising by six time from 2017 to 2019. Online registration completeness was highest in Province 1 (61.2%), followed by Bagmati (45.1%) and Gandaki (45.1%). However, completeness is much lower in Karnali (13.2%) and Sudurpaschim (15.1%). Completeness of under-five online death registration is only 1% for both males and females (Additional File 2: Table A2). Completeness, as measured by the ratio of online to GBD estimated deaths, increased with older age with the exception of a large spike in completeness for adult males, where it peaked at 62% at 35-39 years which was more than double the figure for females of that age group and twice the all-age completeness estimate for both sexes (Additional File 2: Figure A2).

**Table 3:** Online death registration completeness (%) by area and sex, Nepal, 2017-2019

Area	Online registration								
	**2017**			**2018**			**2019**		
	**Both Sexes**	**Male**	**Female**	**Both Sexes**	**Male**	**Female**	**Both Sexes**	**Male**	**Female**
**Nepal (Census Projection)**	18.6	18.9	21.2	25.7	26.8	27.9	31.9	33.9	33.1
**Nepal (UN WPP Population)**	19.3	20.1	21.8	26.7	29.0	28.3	33.0	36.8	33.3
**Nepal (GBD Population)**	18.3	18.5	21.3	24.9	25.8	27.3	30.5	32.6	31.8
**Ecological Belts**									
Mountain	6.3	6.9	7.3	7.4	7.9	8.5	35.0	39.2	32.9
Hill	20.2	20.3	23.2	26.3	27.2	29.0	30.1	31.5	32.1
Terai	20.9	21.1	24.0	30.3	31.4	32.8	39.9	42.0	40.7
**Provinces**									
Province 1	33.1	33.4	37.2	49.2	50.4	51.6	61.2	62.5	62.5
Province 2	15.1	15.4	17.9	17.1	17.4	20.1	20.0	20.7	22.7
Bagmati	30.6	31.2	33.2	40.2	41.4	41.6	45.1	46.7	46.0
Gandaki	21.9	21.9	25.5	34.1	35.7	36.6	45.1	48.4	45.5
Lumbini	16.1	16.0	19.5	24.5	25.4	27.4	38.6	41.5	39.3
Karnali	10.9	10.3	14.7	12.6	12.1	16.6	13.2	12.9	17.1
Sudurpaschim	10.1	11.2	11.2	12.9	14.9	13.5	15.1	17.7	15.4

Note: Completeness for ecological belts and provinces calculated using Census projected population

### Completeness of CRVS Survey reported deaths

The completeness status of reported deaths in CRVS Survey for 2014 and 2015 are provided in Table 4. To avoid the impact of mortality shock, CRVS Survey death reporting completeness estimates are not presented for areas which were severely affected by earthquake in 2015 - Mountain and Hill ecological belts and Bagmati province. In 2014, death reporting completeness was 54%, and just above 50% according to the other two sources of population data. In 2015, the CRVS Survey death reporting completeness was significantly higher at 75%, with again similar levels according to UN WPP and GBD population. The 2015 completeness for males and females was similar, although in 2014 it was somewhat higher for males.

**Table 4:** CRVS survey all-age death reported completeness (%), Nepal by area and sex, 2014-15

Area	CRVS Survey					
	**2014**			**2015**		
	**Both Sexes**	**Male**	**Female**	**Both Sexes**	**Male**	**Female**
**Nepal (Census Projection)**	54.4	57.2	52.9	74.9	74.0	75.9
**Nepal (UN WPP Population)**	56.7	61.6	53.4	77.2	78.7	76.1
**Nepal (GBD Population)**	53.6	55.9	52.8	73.5	72.8	74.7
**Ecological Belts**						
Mountain	45.5	50.3	41.4	-	-	-
Hill	57.6	63.1	52.6	-	-	-
Terai	55.5	54.9	57.9	68.9	67.7	71.0
**Provinces**						
Province 1	60.1	62.6	59.4	76.6	73.6	80.4
Province 2	67.3	63.1	72.8	72.0	66.8	78.1
Bagmati	50.8	53.6	49.0	-	-	-
Gandaki	59.8	65.1	55.9	80.7	83.1	78.2
Lumbini	50.8	56.0	47.2	70.1	72.7	67.6
Karnali	67.3	75.4	56.1	80.3	82.5	76.6
Sudurpaschim	45.6	49.1	44.2	61.0	63.2	59.9

At the subnational level, death reporting completeness was higher in Hill and Terai than Mountain in 2014, the only year where such a comparison could be made. There was relatively smaller variation according to provinces when compared with offline registration, with death reporting completeness ranging from 61% in Sudurpashchim to 81% in Gandaki. Female completeness was higher than for males in Provinces 1 and 2. Completeness of under-five death reporting is 25.0% for males and 17.1% for females (Additional File 2: Table A2).

## Discussion

This study has assessed the completeness of the two major routine sources of mortality data in Nepal at the national and subnational levels – the offline and online death registration systems – as well as the nationally representative CRVS Survey. We found that the offline registration system and CRVS Survey generated mortality data of a reasonable but still suboptimal level of completeness nationally (69% in 2017 and 75% in 2015, respectively), while the online registration system has a considerably lower level of completeness of just 32%. The lack of improvement over time in the completeness of the offline registration data suggests that significant investments in the system are needed to attain a higher level of completeness but may also reflect the inherent weaknesses of the offline system, particularly that it is paper based. Completeness of online death registration, in contrast, almost doubled from 2017 to 2019, however this source is still too incomplete to use for mortality estimation purposes.

A notable finding from the offline registration system is that male completeness is 8% points higher national than for females, reaching over 30% points in Sudurpashchim. Further, in the online registration system, completeness among male adults is far higher than for female adults, being more than double at 35-39 years, although these differences are not found at older ages. There is an existing tradition of male-dominant property ownership in Nepal and provision of social security allowance for widows, and the legal requirement of death registration certificate for transferring the property ownership and getting access to the social security are the major possible reasons behind this difference, particularly for adult deaths [23, 30]. These differences do not exist for the CRVS Survey, which is based on the reporting of deaths by households and not affected by these incentives.

Subnational geographic differences in offline registration are significant, with completeness reaching 90% in Gandaki but just 39% in Karnali. These subnational differences are consistent irrespective of the under-five mortality rate used as an input into the empirical completeness method. Online differences also exist, with higher completeness in Province 1, but particularly low in Province 2, Karnali and Sudurpashchim. There are a range of potential reasons for differences in offline registration completeness. Poor geographic accessibility to a registry office can determine the ability of a family to register a death; consistent with this, a recent study found that geographic accessibility to primary health facilities in Nepal is lowest in the remote Mountain belt, which has a completeness consistently lower than the national level.[31] Karnali Province contains the most remote and mountainous districts and so has low levels of death registration completeness. However, geographic accessibility is not the sole determinant, because in 2017 Terai had the lowest completeness of the ecological belts despite being relatively densely populated. Socio-economic characteristics are also important. Bagmati and Gandaki, which are consistently among the provinces with the highest completeness, are comparatively more developed, have big urban centres including Kathmandu, Pokhara and Biratnagar and are ranked 1st and 2nd in the Human Development Index (HDI) according to the recent Human Development Report 2020 [32]. Sudurpashchim, Karnali and Province 2 consistently have the lowest completeness and also are the lowest ranked in the HDI. Other likely influencing factors are cultural beliefs (which might be reflected in the HDI rankings) and which can discourage certain groups in specific areas from registering deaths, lack of awareness and effective outreach programs, and Government and NGOs targeting civil registration promotion programs in certain subnational populations.

Subnational differences in online registrations may be more related to the uptake of the new system by districts within each province or ecological belt. The rapid transition to the more modern online registration system has occurred for several reasons. Capacity building initiatives were conducted for local registrars’ offices to switch from the offline to online registration system. Due to recent decentralisation, which have made local governments more independent and able to mobilize resources and make decisions, these local offices are playing an important role for introducing online registration systems, especially in specific areas. The growth was initially limited to the urban and developed local areas because more remote areas had limited access to the internet and electricity, however with infrastructure development this is now gradually extending to remote areas. Enhanced collaboration of the Government with different stakeholders, including the World Bank for national CRVS system development is another potential reason behind this growth. Increased mobile phone user numbers has also encouraged people to choose the online registration system, because it enables them to notify the event in online system and progress the registration process. Further detail of these changes is provided in Additional File 1.

A limitation of the analysis of completeness of the offline registration data is that it is reliant upon reporting of deaths by year of registration, however, at the national level at least, the consistency of completeness over the study period suggests that there are no significant differences in delayed registration over time. The completeness calculated based on the GBD and UN WPP estimated deaths is slightly lower than according to the empirical completeness method, but it should be borne in mind that there is some uncertainty about those estimates as they are based on limited local data (primarily early age mortality) and model life tables. Due to the empirical completeness method providing plausible national completeness estimates, it provides confidence in its subnational estimates as well. A further drawback of offline registration data is the lack of age at death data, however the empirical completeness method could still estimate completeness despite its absence preventing us assessing age-specific completeness from this source.

Another limitation is related to the CRVS survey-based death reporting completeness, which was much higher in 2015 than 2014. This may be due to recall bias, i.e. missing reporting of older deaths than more recent deaths. Estimation of completeness of offline registration and CRVS Survey data are also affected by the earthquake in 2015, which meant we could not estimate completeness in 2015 in Mountain and Hill regions and Bagmati province. Further, the offline registration and CRVS Survey reference period was based on the Nepalese calendar, and required conversion to the Gregorian calendar years. Hence, for the CRVS Survey we could not produce results for calendar years 2013 and 2016. The survey was designed using old administrative boundaries but sensitivity analyses revealed its provincial-level estimates to be reliable. Finally, while there is some uncertainty about population estimates due to them being projections from the 2011 Census, the impact of the population data source used on the completeness estimates at the national level was minimal; further, there was no alternative population data source at the subnational level.

## Conclusions

The continued deployment of the online registration system, which has been initiated by only 40% of local level wards in Nepal, should help improve not only the level of completeness of registration but the availability of timely data by age at death and year of occurrence that aren’t presently available in the offline system.[33] Further development of the online system to cover the whole of the country should be the focus of future CRVS developments in Nepal. In the interim, to provide age at death data in the offline system there could be more staff deployed to focus on improving the detail of deaths reported, training and incentives provided to local registrars in reporting age at death, and improvement in the stability of registrars’ positions to reduce high staff turnover. Another major area of improvement is to report valid cause of death data, which should be a primary output from a CRVS system to inform policy. A recent amendment to the death notification form has a provision to report the cause of death only if it has been certified by a physician using the International Form of Medical Certificate of Cause of Death, however until now this is only being implemented in a limited number of hospitals. There should also be focus on improving completeness and quality of medical certification of cause of hospital deaths, capturing cause of community deaths using verbal autopsy, and developing a sustainable mechanism to feed cause of death data compiled from both sources into the CRVS system. Although there are significant areas of improvement needed in Nepal’s CRVS system, the fact that over two-thirds of deaths are registered means that mortality indicators at the subnational level can be developed using demographic techniques applied to these routine data.

The results of the CRVS Survey, which collected data on barriers to death registration based on a nationally representative survey of deaths (measured as 75% complete), will inform these improvements. A comprehensive assessment is needed to identify the major bottlenecks behind this low completeness to attain optimum civil registration coverage by the end of CRVS Decade 2015-2024 [34]. Targeted program intervention for poorly performing areas and groups with low death registration completeness, building strong coordination mechanism among CRVS stakeholders, and implementing public awareness programs are areas that can be focused on. Further studies are planned to address these issues and demonstrate the utility of the vital statistics component of the CRVS system as a source of important reliable, timely and continuous demographic and health indicators that can be used by local analysts and reduce the reliance on estimates made using sparse local data.

## Supplementary Information

**Additional file 1.**

**Additional file 2.**

## Data Availability

Request for data access should be directed to the corresponding author and will be granted subject to approval by the Department of NID and Civil Registration and Central Bureau of Statistics, Nepal.

## Declarations

We declare that the content of this manuscript has not been published or submitted for publication elsewhere.
