# Supplementary material for: Assessment of the national and subnational completeness of death registration in Nepal
Source: BMC Public Health. 2022 Mar 4;22:429. doi: 10.1186/s12889-022-12767-z (PMC8895765; doi:10.1186/s12889-022-12767-z)
Supplement: Supplementary file 1 — Additional file 1. [file 12889_2022_12767_MOESM1_ESM.docx]

**Additional File 1**

**Adjustment for data published according to Nepali calendar**

The offline and online registration and CRVS Survey data were published according to the Nepali (Bikram Sambat) calendar, for which the year commences in mid-April. We converted data to the Gregorian calendar year for all our analyses with deaths allocated according to the proportion of days in the Nepali Calendar in each Gregorian calendar year. This did exclude the latest mortality data according to the Nepal calendar, but this process was the most prudent to ensure consistency of periods with other estimates of mortality by the GBD and UN. The offline registration data is based on year of registration rather than year of occurrence, so annual completeness estimates could be affected by inconsistent levels of late registrations across the years.

**Estimation of under-five mortality rates and population**

The *5q0* at the national level is that published by the United Nations Inter-Agency Group for Child Mortality Estimation (IGME), which is estimated based on statistical modelling of trends based on various sources of data (1). Subnational estimates of *5q0* were made for each province and ecological zone as using 2011 Population Census summary birth history data of children ever born and ever died using the maternal cohort method and 2016 Demographic and Health Survey (DHS) detailed birth history data (1, 2). The average *5q0* of the Census and DHS was calculated and its ratio to the average national *5q0* estimate from those sources was used to scale subnational *5q0* to the national estimate made by the IGME for each year. Another source of *5q0* data is the MICS, but it did not report this indicator by subnational area. Population data at the national level were derived from the GBD, UN WPP and the Nepal Census projections, and we compared the estimated completeness based on these three population data sources to assess the sensitivity of results to the source. Based on census based population projections made by CBS, we interpolated total population at the national level by calculating the exponential growth rates for 2011-16 and 2016-21, and for each age using linear interpolation of the proportion of the population at that age using the same time periods (3). Subnational level total population was estimated by interpolation of total population using exponential growth rates for projected population for two quinquennium 2011-16 and 2016-21 (4). Then we estimated population for each age using the ratio of the proportion of the population at that age in the subnational level compared to the national level and adjusted final estimates by ensuring the sum of the population at all ages equal to the estimated total population.

**Sample design for CRVS Survey**

The CRVS Survey was nationally representative and administered within 80,000 sampled households (1.5% of total 5.4 million total households). For the purpose of sampling, the whole country was divided into 16 analytical domains, including comprising 15 eco-development regions and Kathmandu Valley. A two-stage probability proportional to size sampling scheme was adopted to select 1,600 enumeration areas in the first stage and 50 households from each enumeration area, resulting in 80,000 households. This accounted for 0.3% sample error at a 95% confidence interval. The second stage was not a completely random selection, because 50 households were selected starting from one corner of each enumeration area. The survey was designed using administrative boundaries that existed prior to the change in political structure of Nepal in 2015 when new provincial boundaries were introduced. We worked with the Central Bureau of Statistics and their correspondence files to generate estimates according to the new provincial boundaries. Sensitivity analyses revealed these provincial-level estimates to be reliable according to the survey design. Also, there was no post enumeration survey conducted to check the non-response rate and other possible enumeration errors.

**Architecture and development of the online registration system**

After 2014’s CRVS ministerial level meeting of the United Nations ESCAP region, the Government of Nepal adopted several crucial steps for CRVS development in the country, including the introduction of the online registration system for civil registration management.

Online civil registration is operated under the integrated Vital Event Registration and Social Protection Management Information System (VERSP MIS), managed by the Department of National ID and Civil Registration. Any personal events, including births and deaths, are notified by the authorised notifier via the VERSP MIS system by submitting an online application and a one-time password (OTP) is sent to their mobile phone. The event is registered after document verification and a 13-digit unique individual identifier is generated for that event. A registration certificate is issued with that unique ID. Further, the entered data are accessed by the central MIS team to view and manage data, produce a civil registration report, and link civil registration data with the social security system. All local registrars’ offices can also access respective local area data, manage them, and generate registration certificates for users and produce local level civil registration reports and they could also be able to generate local vital statistics in future. The central office compiles online data from all local registration offices, stores them, and produces and disseminates civil registration reports jointly with paper-based civil registration report. No vital statistics have yet been produced using either of the data. Further information on the architecture is shown in Figure A1.

**Figure A1: Architecture of online event registration system in Nepal**


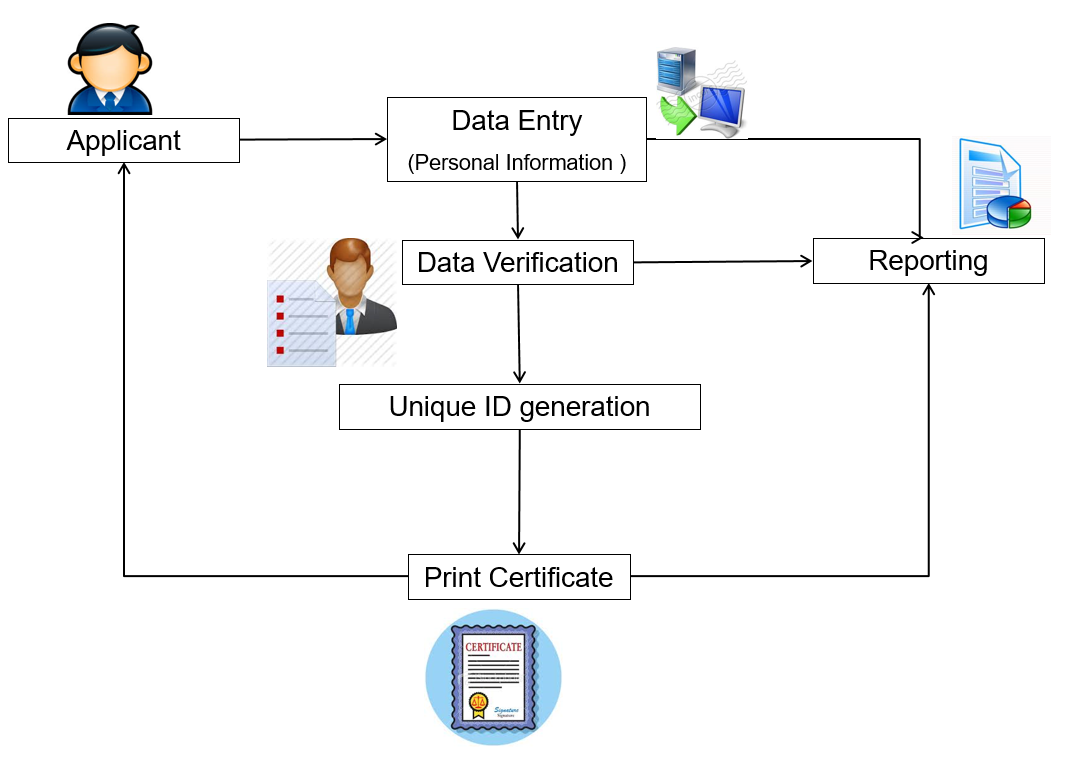


Source: DONIDCR, Nepal

To promote the spread of the online registration system, local registrars’ offices were gradually encouraged to switch from the offline registration system to online registration system by building their capacity. The central civil registration department undertook capacity assessments of different local offices regarding the assessment to basic services, including internet and power supply with availability of physical infrastructure, and trained human resources to implement the online registration system. Due to the importance and effectiveness of online registration system, it was local registrars’ offices that initiated to introduce online registration system. However, due to geographical remoteness and limited access to the internet and electricity, the growth was initially limited to the urban and developed local areas and now gradually extending to remote areas as well.

Recently, a new government department has been formed by merging the existing Department of Civil Registration and the National ID Centre, and existing civil registration legislations have also changed. This change was undertaken to link the unique individual identifier generated via civil registration system with the National ID, and has acted to increase online death registration. Also, increased numbers of mobile phone users at local levels also encouraged people to choose the online registration system, because it requires mobile phone to get the OTP to notify the event in online system and proceed for further registration process.

**Global Burden of Disease and United Nations World Population Prospects methods to estimate total deaths**

The GBD estimates total deaths by developing complete life tables after separately measuring under-five mortality (5q0) and adult mortality (45q15) (5). Under-five mortality is estimated from registration data and summary and complete births histories in censuses and surveys. Annual estimates are generated using spatio-temporal Gaussian process regression that corrects for source-specific bias. The only source of adult mortality data in Nepal that the GBD used is from sibling survival data in the Demographic and Health Survey. Using these estimates of adult mortality, spatio- temporal Gaussian process regression estimates 45q15 over time using socio-economic and regional covariates. Complete life tables are generated from model life tables that use 5q0 and 45q15 estimates as inputs, along with a standard life table which a regional life table for Nepal. The UN World Population Prospects also estimates 5q0 from complete and summary birth histories from censuses and surveys, while only considers household death data from censuses for 45q15. It estimates full life tables by inputting just 5q0 into model life tables (6).

1. UN Inter-agency Group for Child Mortality Estimation, [Internet]. Unicef. 2019 [cited 25/04/2020]. Available from: https://childmortality.org/.

2. Rajaratnam JK, Tran LN, Lopez AD, Murray CJ. Measuring under-five mortality: validation of new low-cost methods. 2010;7(4):e1000253.

3. Shryock HS, Siegel JS, Larmon EA. The methods and materials of demography: US Bureau of the Census; 1973.

4. Central Bureau of Statistics. National Population and Housing Census 2011 Population Projection (2011-2031). Kathnamdu; 2014.

5. G.B.D. Demographics Collaborators. Global age-sex-specific fertility, mortality, healthy life expectancy (HALE), and population estimates in 204 countries and territories, 1950-2019: a comprehensive demographic analysis for the Global Burden of Disease Study 2019. Lancet 2020; 396: 1160-203.

6. United Nations. World Population Prospectus 2019, New York: United Nations 2019 [Available from: https://population.un.org/wpp/DataSources/524].
