# Supplementary material for: Assessment of the national and subnational completeness of death registration in Nepal
Source: BMC Public Health. 2022 Mar 4;22:429. doi: 10.1186/s12889-022-12767-z (PMC8895765; doi:10.1186/s12889-022-12767-z)
Supplement: Supplementary file 2 — Additional file 2. [file 12889_2022_12767_MOESM2_ESM.docx]

**Additional File 2**

**Additional Tables and Figures**

**Table A1: Mortality data availability status in Nepal**

| **Data source** | **Year/s mortality data available** | **Type mortality data** | **Annual number of deaths** | **Data by age?** | **Subnational levels**** |
| --- | --- | --- | --- | --- | --- |
| Registration – offline (paper) | 2012/13-2019/20 | Registered deaths | 114,436 (2017) | No | Provinces, ecological belts |
| Registration – online | 2019 | Registered deaths | 49,026 (2019) | Yes | Provinces, ecological belts |
| CRVS survey | 2013/14- 2015/16 | Reported deaths in last 12 months, whether deaths registered | 126,292 (2015) | Yes | Provinces, ecological belts |
| Census | 2010-11 | Reported deaths in last 12 months | 129,978 (2010-11) | Yes | Provinces, ecological belts |

*****Online registered deaths for Nepali year of 2075 (2018/19)

** Provinces level refers to the seven new provinces formed in 2015.

**Table A2: Under five death reporting completeness by geography and sex: Online death registration (2017-2019) and CRVS Survey (2014-2015)**

| **Area** | **Online Death Registration** | | | | | | **CRVS survey** | | | |
| --- | --- | --- | --- | --- | --- | --- | --- | --- | --- | --- |
|  | **2017** | | **2018** | | **2019** | | **2014** | | **2015** | |
|  | Male | Female | Male | Female | Male | Female | Male | Female | Male | Female |
| **Nepal** | **0.5%** | **0.5%** | **0.7%** | **0.7%** | **1.1%** | **1.0%** | **23.8%** | **16.3%** | **25.0%** | **17.1%** |
| Mountain | 0.1% | 0.2% | 0.2% | 0.2% | 0.9% | 0.8% |  |  | 75.4% | 51.6% |
| Hill | 0.6% | 0.6% | 0.8% | 0.8% | 1.1% | 1.0% |  |  | 35.8% | 27.4% |
| Terai | 0.6% | 0.4% | 0.9% | 0.9% | 0.9% | 0.9% |  |  | 38.6% | 21.6% |
| Province 1 | 1.7% | 1.1% | 2.1% | 1.4% | 3.0% | 3.1% |  |  | 22.7% | 18.8% |
| Province 2 | 0.1% | 0.0% | 0.2% | 0.3% | 0.6% | 0.3% |  |  | 33.7% | 23.0% |
| Bagmati | 0.7% | 0.7% | 0.9% | 1.1% | 1.5% | 1.1% |  |  | 59.5% | 41.5% |
| Gandaki | 0.9% | 1.7% | 1.4% | 1.2% | 2.1% | 2.4% |  |  | 23.8% | 19.6% |
| Lumbini | 0.3% | 0.5% | 0.7% | 1.1% | 1.3% | 1.5% |  |  | 13.5% | 10.8% |
| Karnali | 0.2% | 0.1% | 0.3% | 0.5% | 0.5% | 0.4% |  |  | 30.1% | 20.3% |
| Sudurpaschim | 0.2% | 0.3% | 0.3% | 0.4% | 0.7% | 0.6% |  |  | 14.6% | 4.3% |

**Table A3: Offline death registration completeness using UNIGME and authors’ under five mortality estimates, both sexes, Nepal, 2017**

| **Provinces** | **Completeness (based on UNIGME 5q0 estimates)** | **Ranking (UNIGME 5q0)** | **Completeness (based on authors’ 5q0 estimates)** | **Ranking (authors’ 5q0)** | **Difference in percentage points (p.p.) (IGME minus authors’ estimates)** |
| --- | --- | --- | --- | --- | --- |
| Province 1 | 74.7% | 4 | 74.6% | 4 | +0.1 p.p. |
| Province 2 | 49.2% | 6 | 54.1% | 6 | -4.9 p.p. |
| Bagmati | 76.4% | 3 | 77.1% | 3 | -0.7 p.p. |
| Gandaki | 90.5% | 1 | 90.2% | 1 | +0.3 p.p. |
| Lumbini | 80.2% | 2 | 77.7% | 2 | +2.5 p.p. |
| Karnali | 45.9% | 7 | 38.6% | 7 | +7.3 p.p. |
| Sudurpashchim | 71.4% | 5 | 63.8% | 5 | +7.6 p.p. |

**Figure A2: Ratio of online registered deaths to GBD estimated deaths by age and sex, Nepal, 2019**
